# Relationships among Different Water-Soluble Choline Compounds Differ between Human Preterm and Donor Milk

**DOI:** 10.3390/nu9040369

**Published:** 2017-04-07

**Authors:** Sara Moukarzel, Lynda Soberanes, Roger A. Dyer, Susan Albersheim, Rajavel Elango, Sheila M. Innis

**Affiliations:** 1 Department of Pediatrics, University of California San Diego, San Diego, USA and Larsson-Rosenquist Foundation Mother-Milk-Infant Center of Research Excellence, Health Sciences, University of California, San Diego, CA 92093, USA; smoukarzel@ucsd.edu; 2 Department of Pediatrics, University of British Columbia, Vancouver, BC V6H 3V4, Canada; lSoberanes@cfri.ca (L.S.); radyer@mail.ubc.ca (R.A.D.); salbersheim@cw.bc.ca (S.A.); mksara@alumni.ubc.ca (S.M.I.); 3 Division of Neonatology, BC Women’s Hospital and Health Centre, Vancouver, BC V6H 3N1, Canada; 4 Origins of Child Health and Disease, Healthy Starts, BC Children’s Hospital Research Institute, Vancouver, BC V5Z 4H4, Canada; 5 School of Population and Public Health, University of British Columbia, Vancouver, BC V6T 1Z3, Canada

**Keywords:** choline, preterm infant, human milk, donor milk, neonatal intensive care, breastfeeding

## Abstract

Choline is essential for infant development. Human milk choline is predominately present in three water-soluble choline (WSC) forms: free choline (FC), phosphocholine (PhosC), and glycerophosphocholine (GPC). It is unclear whether mother’s own preterm milk and pooled donor milk differ in WSC composition and whether WSC compounds are interrelated. Mother’s own preterm milk (*n* = 75) and donor milk (*n* = 30) samples from the neonatal intensive care unit, BC Women’s Hospital were analyzed for WSC composition using liquid chromatography tandem mass spectrometry (LC-MS/MS). Associations between different WSC compounds were determined using Pearson’s correlations, followed by Fischer r-to-z transformation. Total WSC concentration and concentrations of FC, PhosC, and GPC did not significantly differ between mother’s own milk and donor milk. FC was negatively associated with PhosC and GPC in mother’s own milk (*r* = −0.27, *p* = 0.02; *r* = −0.34, *p* = 0.003, respectively), but not in donor milk (*r* = 0.26, *p* = 0.181 *r* = 0.37, *p* = 0.062, respectively). The difference in these associations between the two milk groups were statistically significant (*p* = 0.03 for the association between PhosC and FC; and *p* = 0.003 for the association between FC and GPC). PhosC and GPC were positively associated in mother’s own milk (*r* = 0.32, *p* = 0.036) but not donor milk (*r* = 0.36, *p* = 0.062), although the difference in correlation was not statistically significant. The metabolic and clinical implications of these associations on the preterm infant need to be further elucidated.

## 1. Introduction

Choline (*N,N,N*-trimethylethanolammonium) was established as an essential nutrient by the Institute of Medicine in 1998, based on evidence of insufficient hepatic de novo synthesis in adults [1]. Recommended intakes for infants were set as Adequate Intake (AI) for *total* choline. AI for infants 0–6 months old was set at 125 mg/day choline, estimated mathematically at 25–31 mg/kg/day for preterm infants ≤1200 g [2]. However, not addressed in current recommendations, human milk provides the infant with various forms of choline. The three water-soluble choline (WSC) compounds free choline (FC), phosphocholine (PhosC), and glycerophosphocholine (GPC) contribute to around 90% of the total choline compounds in milk. The lipid-soluble phosphatidylcholine and sphingomyelin account for the remaining 10% [3,4,5,6]. Choline per se has crucial roles in lipid synthesis and transport, neurotransmission, and methylation, which importantly involve choline in different forms *in the body* [7]. Earlier work by Cheng et al. (1996) showed that differences exist in the bioavailability and metabolism between the different WSC compounds in rat milk [8]. In light of these differences, Lewis et al. (2015) have recently proposed the need for studies to address the forms of choline consumed in the diet, in addition to total choline intake, when exploring the implications of choline for health [9]. 

There is an increase in banking of donor human milk for consumption by other infants when mother’s own milk is insufficient in volume or is unavailable, especially in hospitals with preterm infants [10]. Donor milk processing protocols typically involve pooling of milk from multiple women at different stages of lactation into one batch, followed by pasteurization and aliquoting into smaller volumes. The concentrations of total and individual WSC compounds in milk vary widely across women [6,11], and total choline concentration in milk seems to decrease with postnatal age [12]. Additionally, milk samples from women who delivered preterm (*n* = 353; milk collected at postnatal age 6–85 days) were recently reported to be significantly lower in total choline compounds, GPC, and PhosC and higher in FC compared to milk from women who delivered at term (*n* = 9; milk collected at postnatal age 6–118 days) [6,12]. We questioned whether preterm infants fed donor milk in our neonatal intensive care unit (NICU) consume milk with different WSC compounds concentrations compared to those fed their mother’s own milk. Therefore, our primary objective was to determine and compare the concentrations of WSC compounds in mother’s own preterm milk and donor human milk fed at the NICU in the British Columbia Women’s Hospital and Health Center. 

Foods differ in the ratios of the different forms of a particular nutrient, and interactions in digestion, absorption, and metabolism between these different dietary forms, including those of vitamins, are not uncommon. In human milk, it is unclear whether the different WSC compounds are interrelated (i.e., Is milk low in one WSC compound high in another compound an indication of WSC synthesis regulation in the mammary gland?). The effects of consuming varying WSC proportions from human milk on infant hepatic and brain metabolism is an active area of research [13]. Given that donor milk is often pooled from multiple donors, we questioned whether relationships between WSC compounds in preterm milk, if present, are maintained in donor milk. This is important to guide the development of human milk processing protocols that result in minimal “distortion” of physiologic WSC composition in human milk, if any. Therefore, our secondary objective was to determine the relationships between various milk WSC compounds and to compare these relationships between preterm milk and donor milk. We found that preterm and donor milk samples were not significantly different in WSC composition. FC was negatively associated with GPC and PhosC in preterm milk, but not in donor milk.

## 2. Materials and Methods

### 2.1. Data Collection

Preterm milk (milk from mothers who gave birth before 37 weeks of gestation) and term donor milk samples were collected daily at the NICU in BC Women’s Hospital and Health Center in Vancouver, British Columbia (BC), Canada, for a duration of one month, between 7:00 a.m. and 9:00 a.m., coinciding with the timing when nurses typically prepared milk feeds. It was standard of practice for the nurse to place the milk vials by the bedside, allowing milk to reach room temperature, before feeding. Once the desired temperature was achieved, indicated by the nurse, milk samples (500 µL) were taken from the vials using single-use sterile syringes, only when the available milk exceeded the volume needed to feed the infants. Samples were placed on ice and transferred immediately to the nutrition laboratory within close proximity to the hospital.

Personal identifiers for the mother or the infant were not collected, and samples were given random codes for laboratory analyses. At the time of this study, milk fed to infants in the NICU was being analyzed for energy and macronutrients as a Quality Improvement project. As only milk volumes that would have been in excess to infant needs were used and all samples were de-identified, individual consent for participation in this study was not considered necessary. Accordingly, no data relevant to maternal and infant demographic and health characteristics that would otherwise require parent consent was collected. Donor milk batches were prepared by pooling milk samples from multiple donors who met several inclusion criteria: are able to provide at least 150 ounces of milk, have infants less than one year of age, have negative results on pre-screening blood tests for infectious diseases (i.e., HIV, syphilis, hepatitis), do not smoke or use tobacco products, do not take any medication on regular basis, and have not used illegal drugs in the last 5 years. Data on the age of infants of milk bank donors whose milk were analyzed in this study were not available, and the historical average infant age associated with all donors collectively is approximately 6 months. This study was conducted according to the guidelines laid down in the Declaration of Helsinki and was approved (H14-00714) by the University of British Columbia—Children’s and Women’s Health Centre of BC Research Ethics Board (UBC C & W REB). 

### 2.2. Biochemical Assessment

Milk samples were immediately analyzed in duplicates for total lipid content (g/dL) using the creamatocrit method [14]. Aliquots for the analysis of total protein (100 µL) and WSC compounds (20 µL) were then taken and samples were stored at −80 °C for future analysis. Total protein content was determined by the Bradford method [15]. FC, PhosC, and GPC were analyzed using isotope dilution liquid chromatography tandem mass spectrometry (LC-MS/MS), using a Waters ACQUITY UPLC system connected to a Quattro Micro tandem MS configured with an electrospray source (Waters Corporation, Milford, MA, USA). The LC included a 2.1 × 12.5 mm pre-column and a 2.1 × 150 mm Zorbax Rx-Silica column, both with 5 µm particle size (Agilent Technologies, Santa Clara, CA, USA). Injection volume was 4 μL. Two mobile phases were used: A = acetonitrile with formic acid (0.1%) and trifluoroacetic acid (0.1%) and B = ammonium formate (15 mmol/L) with formic acid (0.1%) and trifluorocetic acid (0.2%) in water. The solvent gradient started with 95% A and 5% B the first 2 min, followed by a linear gradient to 55% A and 45% B at 4 min, maintained until the end of the run such that the total analytical time was 9 min. Flow rate was maintained at 0.5 mL/min. The LC column and autosampler were maintained at 25 °C and 5 °C, respectively. The MS was operated in positive ion multiple reaction monitoring (MRM) mode using the following transitions (*m*/*z* (compound)): 103.9/59.9 (choline), *m*/*z* 113/68.9 (choline-d9), 184.1/125.0 (PhosC), 193.1/125.0 (PhosC-d9), 258/124.9 (GPC), 289.0/221.0 (GPC-d9). Based on earlier methods development in our laboratory, the analytical inter-assay and intra-assay coefficient of variations (CVs) for each of the WSC compounds were as follows: for PhosC, 6.4% and 5.2%, respectively; for FC, 5.5% and 4.1%, respectively; and for GPC 9.5% and 2.3%, respectively [8]. Intra-assay CV was calculated on five replicates of the same sample done on one day, all processed at the same time. Inter-assay CV was calculated on aliquots of the same sample analyzed once per day for five separate days.

### 2.3. Statistical Analysis

The normality of data distribution for milk lipid content (g/dL), milk protein content (g/dL), and concentrations of milk FC, PhosC, and GPC (µmol/L) was tested using Kolmogorov-Smirnov test. The distributions for milk lipid content in both mother’s own and donor milk as well as GPC and FC in mother’s own milk were skewed and differences between preterm and donor milk samples were analyzed using Mann Whitney U test. For all other variables, data was normally distributed and independent samples *t*-tests were used. Descriptive statistics were used to identify outliers. Three data points corresponding to preterm milk GPC concentrations of 1813, 1718, and 1046 μmol/L were identified as outliers. For PhosC in preterm milk, two outliers with concentrations of 1969 and 1775 μmol/L were identified. We re-analyzed the corresponding milk samples and confirmed that these values are accurate and therefore retained them in subsequent analyses. The associations between WSC compounds in milk were analyzed in each type of milk, separately, using Spearman’s rank correlation coefficient for preterm milk and Pearson’s correlation coefficient for donor milk, initially. Fischer r-to-z transformation was then used to determine whether differences in correlation coefficients between preterm and donor milk were statistically significant [16]. Statistical analyses were done using SPSS version 21 (SPSS Inc.; Chicago, IL, USA), and a *p*-value < 0.05 was considered statistically significant. 

## 3. Results

A total of *n* = 219 preterm milk and *n* = 45 donor milk samples were collected in the original study, from which *n* = 75 preterm milk and *n* = 30 donor milk samples had remaining volumes available for choline analysis. Preterm milk samples were from mothers of infants 31 (6–39) (median (range)) days old at time of data collection. The total lipid content had wider variability among preterm milk (3.02 ± 1.54 g/dL; median: 3.02; range: 1.30–6.79) than donor milk samples (2.76 ± 0.98 g/dL; median: 2.68; range: 1.10–4.77). Preterm milk samples were significantly higher in total lipid (*p* = 0.03) and protein content than donor milk (3.5 ± 0.5; 3.2 ± 0.4 g/dL, respectively; *p* < 0.001). 

Milk fed at our NICU contained variable amounts of WSC compounds, with no significant differences in total and individual WSC compounds between preterm and donor milk samples (Table 1). 

PhosC and GPC, not free choline, were the major forms of choline in milk. On average, PhosC contributed to 57% of the total WSC compounds in both preterm and donor milk samples, followed by GPC which contributed to 27% and 30% of the total WSC compounds in preterm and donor milk samples, respectively. FC accounted for the remaining 16% and 13% of WSC compounds in preterm and donor milk samples, respectively. Ranges are presented in Table 1 to highlight the wide variability in WSC compounds across samples. 

**Figure 1:**
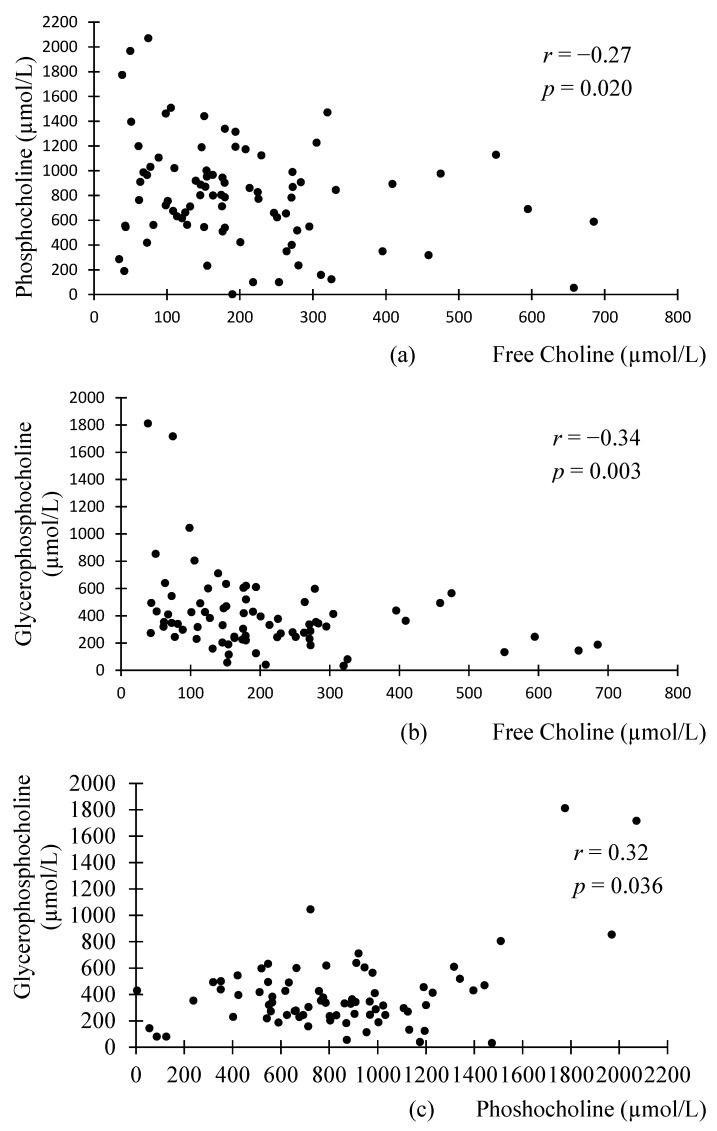
Scatterplots of the relationship between the different water-soluble choline compounds in preterm milk: (**a**) significant negative association between phosphocholine and free choline in preterm milk; (**b**) significant negative association between glycerophosphocholine and free choline in preterm milk; (**c**) significant positive association between glycerophosphocholine and phosphocholine in preterm milk. *n* = 75; associations were analyzed using Pearson’s Correlation.

**Figure 2:**
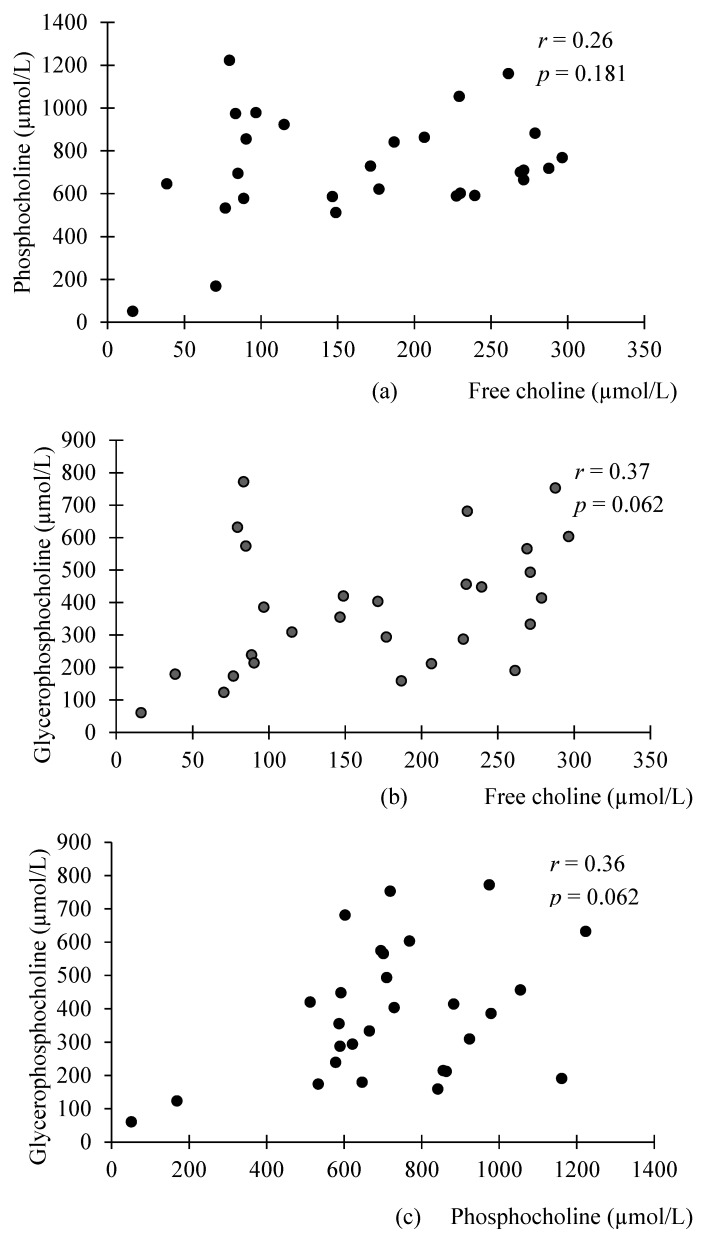
Scatterplots of the relationship between the different water-soluble choline compounds in term donor milk: (**a**) no significant association between phosphocholine and free choline in term donor milk; (**b**) no significant association between glycerophosphocholine and free choline in term donor milk; (**c**) no significant association between glycerophosphocholine and phosphocholine in term donor milk. *n* = 30; associations were analyzed using Spearman’s Correlation.

Interestingly, significant correlations were found between the different WSC compounds in preterm milk, but not donor milk (Figure 1 and Figure 2). FC was negatively associated with PhosC and GPC in preterm milk (*r* = −0.27, *p* = 0.02; *r* = −0.34, *p* = 0.003, respectively), but not in donor milk (*r* = 0.26, *p* = 0.181 *r* = 0.37, *p* = 0.062, respectively). Although these associations did not reach statistical significance in donor milk samples, we note that the direction of associations is opposite to those in preterm milk. Accordingly, the difference in correlation coefficients between preterm and donor milk samples were statistically significant (*p* = 0.03 for the difference between preterm and donor milk regarding the association between PhosC and FC and *p* = 0.003 for the difference between preterm and donor milk regarding the association between FC and GPC). Additionally, PhosC and GPC were significantly positively associated in preterm milk (*r* = 0.32, *p* = 0.036), but not donor milk (*r* = 0.36, *p* = 0.062), although the difference in correlation coefficient between the two groups of milk was not statistically significant (*p* = 0.68). 

## 4. Discussion

In this study, we report that the WSC concentrations of donor milk fed to infants in BC Women's Hospital NICU do not significantly differ from that of mother’s own preterm milk. Our data show an inverse relationship between choline in free form and the bound-choline forms GPC and PhosC in preterm milk only, suggesting a potential physiologic interaction among individual WSC compound de novo synthesis or uptake into the mammary gland (only if the relationship is also present in milk from healthy women delivering at term). However, in donor milk this relationship does not seem to exist, which may be partly explained by the fact that we only analyzed *n* = 30 donor milk samples compared to *n* = 75 preterm milk samples. Alternatively, this may be explained by the pooling of milk from various donors to produce a larger batch of pasteurized donor milk. Additionally, total WSC concentration seems to vary across lactation [12] and a wide difference in lactation age between the two milk groups may be a contributing factor. We are limited by lack of data on the age of infants at the time their mothers donated milk used in our study. However, mothers were eligible to donate to the milk bank if their infants were less than one year of age and the historical average is 180 days, compared to 31 (range: 6–39) days for the mother’s own milk samples. Additionally, mothers were eligible if they were able to donate at least 150 US ounces (4.44 L) of milk. WSC composition in donor milk samples may therefore reflect composition of milk when abundantly produced later in lactation, compared to milk produced earlier in lactation. If confirmed in future studies, the lack of this significant relationship among WSC compounds in donor milk raises the question of what the dietary, metabolic, and clinical implications of these associations are for the preterm infant, when they exclusively receive donor milk, due to the lack of mother’s own milk. 

Consistent with previous studies [6,11,12], PhosC and GPC were the major choline forms in both preterm and donor milk samples, and the proportions of WSC compounds did not seem to vary by milk group. In our study, PhosC ranged between 57%–59%, GPC between 27%–30%, and FC between 13%–16% of the total milk WSC compounds. Of note, comparisons between studies regarding the proportions of various WSC compounds in milk should be addressed with caution because studies vary in the number and nature of milk choline compounds measured. For example, milk PhosC in our study seems much higher than previously reported (~45%) [6,12]. However, here we report % of *total WSC compounds,* whereas others reported *% of total choline compounds* (WSC compounds plus lipid-soluble compounds).

Choline has a wide array of functions that support infant development. Choline, an essential component of phosphatidylcholine and sphingomyelin, is involved in maintenance of cell membrane structural integrity and signaling pathways [7]. In the fast-growing preterm infant, a high demand for choline is required, not only to support cell membrane maintenance, but also to support parenchymal growth, cell proliferation, and membrane formation [17,18]. Choline, via its oxidized form betaine, is involved in donation of methyl groups (CH_3_) particularly for the generation of *S*-adenosylmethionine (SAM) [7,19,20]. SAM is pivotal for more than 100 biochemical reactions such as for glutathione and creatine synthesis and regulation of cellular differentiation and apoptosis [19,20]. Additionally, choline is crucial for brain function, as a precursor of the neurotransmitter acetylcholine [7,19]. However, the contribution of different choline forms in milk to the different choline functions is largely unknown. Interestingly, plasma FC concentrations in preterm infants receiving nutritional support in the NICU (*n* = 56) are around half the concentrations found in cord blood of newborns (*n* = 176) at equivalent gestation ages of 24–42 weeks [21]. As suggested by Bernhard and colleagues [2,21], it is possible that the lower plasma FC concentration in preterm infants indicates a potential necessity for higher *total* choline dietary supply to sustain rapid growth in preterm infants. We speculate that the form of dietary choline is also important for meeting infant requirements for different choline functions [13]. Indeed, plasma FC concentrations in formula-fed term infants are around half those of breast-fed term infants (10.8 ± 2.42; 21.8 ± 7.61 μmol/L, respectively) [5]. In this study, the authors reported significant positive associations between infant FC concentrations in serum and FC, GPC, and PhosC concentrations in consumed human milk. No significant associations were found between other choline compounds in serum (e.g., GPC, PhosC) and choline compounds in milk. Although the authors did not correlate serum choline concentrations in the formula-fed group with WSC choline concentrations in consumed formula, the term infant formula products contained variable concentrations of FC, PhosC, and GPC. The latter had significant differences with concentrations of FC, PhosC, and GPC in the term human milk samples. Accordingly, we raise the question about whether the difference in serum FC concentrations between breastfed and formula fed infants is explained by differences in the total intake of GPC, PhosC, and FC.

To our knowledge, our study is the first to explore the relationship between individual WSC compounds in human milk generally, and in milk of women delivering preterm specifically. This is the first step in generating additional hypotheses about the maternal physiologic determinants of WSC composition in milk and ultimately about which milk composition comprises adequate infant choline intake. Little is known about the uptake and de novo synthesis of WSC by the human mammary epithelial cells. In rats, FC is actively transported into the mammary epithelium within serum FC concentration ranges found in humans [22]. Similar to these results in rats, Ilcol et al. [5] reported a positive association between FC concentrations in term human milk and in maternal serum (*r* = 0.541, *p* < 0.001). However, this does not seem to be the case for GPC and PhosC, whose concentrations in human milk were not related to the concentrations of GPC, PhosC, FC, and lipid-bound choline in maternal serum [5]. Alternatively, the mammary gland can synthesize phosphatidylcholine (PC) via the CDP-choline pathway, using FC as a precursor [23]. PhosC is an intermediate in this pathway (phosphorylation of FC into PhosC by choline kinase), and PC may be used to produce GPC, first by breakdown into lyso-PC and then into GPC. Therefore, we speculate that the lower FC in milk may be secondary to the use of FC for GPC and PhosC synthesis. Research regarding the regulation of PC synthesis within the mammary gland is lacking. A better understanding of the metabolic regulation of PC synthesis in the mammary gland may shed light on WSC synthesis regulation. Importantly, studies that improve our understanding of why the mammary gland invests energy producing choline compounds in different forms will answer whether pediatric clinical nutrition should be focused on the quality of choline intake (i.e., composition of choline compounds) rather than quantity (total choline intake) alone. Recent implications of dietary choline in gut microbiome composition, which has the potential to impact host health [24,25], is by itself an intriguing finding to prompt research on potential distinct biological functions of WSC compounds in preterm human milk.

Due to the secondary analysis nature of this study, clinical and dietary data were not available to explore the impact of maternal factors on preterm milk WSC composition. We also had one milk sample per mother, which only provides cross-sectional data on WSC composition and does not account for the well-known intra-individual variability in milk choline composition [4,11]. Previous findings on positive associations between dietary choline (from food and supplements) and choline composition in term milk suggest maternal choline intake during pregnancy may explain WSC composition in preterm milk [3,11]. Additionally, dietary choline has been shown to be negatively associated with markers of inflammation and markers of oxidative stress in human plasma [26,27]. Interestingly, proinflammatory and oxidative stress pathways are implicated in preterm labor and delivery [28,29]. We recommend that future studies address the role of maternal diet and clinical status in preterm milk WSC composition.

## 5. Conclusions

In conclusion, our findings suggest pooled donor milk fed in our NICU does not significantly differ in WSC composition (total concentration and proportions of WSC compounds) compared to mother’s own preterm milk. However, FC was negatively associated with GPC and PhosC in preterm milk, but not in donor milk, suggesting that physiologic interactions among individual WSC compound de novo synthesis or uptake into the mammary gland may be different in preterm versus donor milk. Future studies with larger sample sizes, particularly for donor milk, are recommended to confirm these findings and to determine biological or environmental factors that explain the relationships (or lack of relationships) between milk WSC compounds. The impact of these differences in preterm infants receiving exclusive donor milk, and how it affects growth and development, needs to be explored further. 

## Figures and Tables

**Table 1:** Concentrations of water-soluble choline compounds in human preterm and term donor milk samples.

µmol/L	Preterm Milk *n* = 75	Term Donor Milk *n* = 30	*p*-Value
Phosphocholine			0.068 ^1^
Mean ± SD	859 ± 385	722 ± 255	
Median	805	705	
Range	3.4–2070	50.8–1223	
Glycerophoshocholine			0.928 ^2^
Mean ± SD	404 ± 294	383 ± 195	
Median	343	370	
Range	33.5–1813	60.5–772	
Free choline			0.569 ^2^
Mean ± SD	203 ± 139	170 ± 86.5	
Median	176	174	
Range	38.4–685	16.3–297	
Total WSC compounds			0.322 ^2^
Mean ± SD	1460 ± 543	1275 ± 414	
Median	1365	1293	
Range	531.3–3863	127.6–1934	

^1^ differences between preterm and term donor milk analyzed by independent samples *t*-test; ^2^ differences between preterm and term donor milk analyzed by Mann-Whitney U test. WSC, water-soluble choline.
